# Elucidating the susceptibility to breast cancer: an in-depth proteomic and transcriptomic investigation into novel potential plasma protein biomarkers

**DOI:** 10.3389/fmolb.2023.1340917

**Published:** 2024-01-18

**Authors:** Yang Wang, Kexin Yi, Baoyue Chen, Bailin Zhang, Gao Jidong

**Affiliations:** ^1^ Department of Breast Surgical Oncology, National Cancer Center/National Clinical Research Center for Cancer/Cancer Hospital, Chinese Academy of Medical Sciences and Peking Union Medical College, Beijing, China; ^2^ Department of General Surgery, Beijing Puren Hospital, Beijing, China; ^3^ Department of Breast Surgical Oncology, National Cancer Center/National Clinical Research Center for Cancer/Cancer Hospital and Shenzhen Hospital, Chinese Academy of Medical Sciences and Peking Union Medical College, Shenzhen, China

**Keywords:** proteome-wide association study, transcriptome-wide association study, plasma proteins, mendelian randomization, breast cancer

## Abstract

**Objectives:** This study aimed to identify plasma proteins that are associated with and causative of breast cancer through Proteome and Transcriptome-wide association studies combining Mendelian Randomization.

**Methods:** Utilizing high-throughput datasets, we designed a two-phase analytical framework aimed at identifying novel plasma proteins that are both associated with and causative of breast cancer. Initially, we conducted Proteome/Transcriptome-wide association studies (P/TWAS) to identify plasma proteins with significant associations. Subsequently, Mendelian Randomization was employed to ascertain the causation. The validity and robustness of our findings were further reinforced through external validation and various sensitivity analyses, including Bayesian colocalization, Steiger filtering, heterogeneity and pleiotropy. Additionally, we performed functional enrichment analysis of the identified proteins to better understand their roles in breast cancer and to assess their potential as druggable targets.

**Results:** We identified 5 plasma proteins demonstrating strong associations and causative links with breast cancer. Specifically, PEX14 (OR = 1.201, *p* = 0.016) and CTSF (OR = 1.114, *p* < 0.001) both displayed positive and causal association with breast cancer. In contrast, SNUPN (OR = 0.905, *p* < 0.001), CSK (OR = 0.962, *p* = 0.038), and PARK7 (OR = 0.954, *p* < 0.001) were negatively associated with the disease. For the ER-positive subtype, 3 plasma proteins were identified, with CSK and CTSF exhibiting consistent trends, while GDI2 (OR = 0.920, *p* < 0.001) was distinct to this subtype. In ER-negative subtype, PEX14 (OR = 1.645, *p* < 0.001) stood out as the sole protein, even showing a stronger causal effect compared to breast cancer. These associations were robustly supported by colocalization and sensitivity analyses.

**Conclusion:** Integrating multiple data dimensions, our study successfully pinpointed plasma proteins significantly associated with and causative of breast cancer, offering valuable insights for future research and potential new biomarkers and therapeutic targets.

## 1 Introduction

In 2020, a concerning 2.3 million women were diagnosed with breast cancer, establishing it as the most common cancer among women worldwide (Sung et al., 2021). This high prevalence underscores the urgency for ongoing research; however, despite significant efforts, the precise causes of breast cancer remain elusive. The disease is marked by a wide range of biological characteristics, including diverse histological and molecular features (Prat and Perou, 2011). Among these, the estrogen receptor (ER) status stands out as a crucial biomarker, significantly influencing treatment strategies such as endocrine therapy for ER-positive breast cancers (Trayes and Cokenakes, 2021). In addition to tissue-specific protein markers, the study of proteins in circulating plasma, often found due to cellular leakage or active secretion (Anderson and Anderson, 2002), is increasingly important. Due to the ease of detection and reproducibility of plasma proteins, these proteins are suitable for biomarkers and potential therapeutic targets (Suhre et al., 2021). Recent studies have highlighted the significant relationship between a variety of circulating proteins and breast cancer, thereby providing crucial insights into the disease’s prognosis (Key et al., 2010; Christopoulos et al., 2015; Rosendahl et al., 2021; Veyssière et al., 2022; Mälarstig et al., 2023). The identification of these proteins as potential biomarkers has opened new avenues for early detection and personalized medicine in breast cancer, emphasizing the importance of understanding the complex biological interactions and pathways involved in cancer progression.

Genome-wide association studies (GWAS) have been instrumental in identifying nearly 200 genetic loci associated with breast cancer, revealing insights into genetic predispositions (Michailidou et al., 2017; Shu et al., 2020; Zhang et al., 2020; Gudjonsson et al., 2022). These discoveries underscore the importance of genetic factors in breast cancer susceptibility. Particularly, SNPs located within a 500 Kb range of the transcription start sites of protein-coding genes, known as cis-acting quantitative trait loci (cis-QTLs). Among these, protein Quantity Trait Loci (pQTLs) are crucial for regulating protein levels and are valuable tools for research (Sun et al., 2018). Utilizing pQTL as genetic proxies allow us to make a deeper exploration of the role of plasma proteins in breast cancer susceptibility. Recently, Proteome-Wide Association Studies (PWAS) (Wingo et al., 2021) and Transcriptome-Wide Association Studies (TWAS) (Gusev et al., 2016) have been pivotal in understanding the functions of proteins and gene expression in disease onset and progression. Initial PWAS focused primarily on neurological contexts due to data limitations (Zhang et al., 2022a), However, recent advancements (Zhang et al., 2022a) have broadened the scope of these studies to include diverse health conditions, thereby enriching our understanding of the associations between plasma proteins and various diseases (Li et al., 2023).

Our first phase focused on identifying proteins that are inherently associated with breast cancer at both proteomic and transcriptomic levels. For PWAS analysis, we integrated plasma protein pQTL data from ARIC cohort (Zhang et al., 2022a) with breast cancer GWAS summary data, including its different ER subtypes. Additionally, we carried out a supplementary TWAS in whole blood and breast mammary tissues. This combined P/TWAS methodology revealed significant associations between plasma proteins and breast cancer. However, it is crucial to note that such associations do not automatically imply causations. To address this, in our second phase, we employed two-sample Mendelian Randomization (MR) analysis (Emdin et al., 2017), adding a causal dimension to the protein-breast cancer relationship. We further assessed shared causal variants between them by genetic Bayesian colocalization. To ensure the robustness and broader applicability of our findings, we further conducted external validations of the established causal link. These validations were achieved using 4 extensive large plasma protein pQTL datasets (Folkersen et al., 2017; Sun et al., 2018; Ferkingstad et al., 2021; Gudjonsson et al., 2022) and the eQTLGen dataset (Võsa et al., 2021).

In our study, we implemented a two-phase design that integrates P/TWAS with MR analyses. This comprehensive methodology, blending associative and causative analyses, provides valuable insights into breast cancer. Furthermore, the relative simplicity in detecting plasma proteins not only strengthens their role in development of diagnostic biomarkers but also suggests their potential value in the development of therapeutic targets for breast cancer.

## 2 Materials and methods

### 2.1 Research framework

**FIGURE 1:**
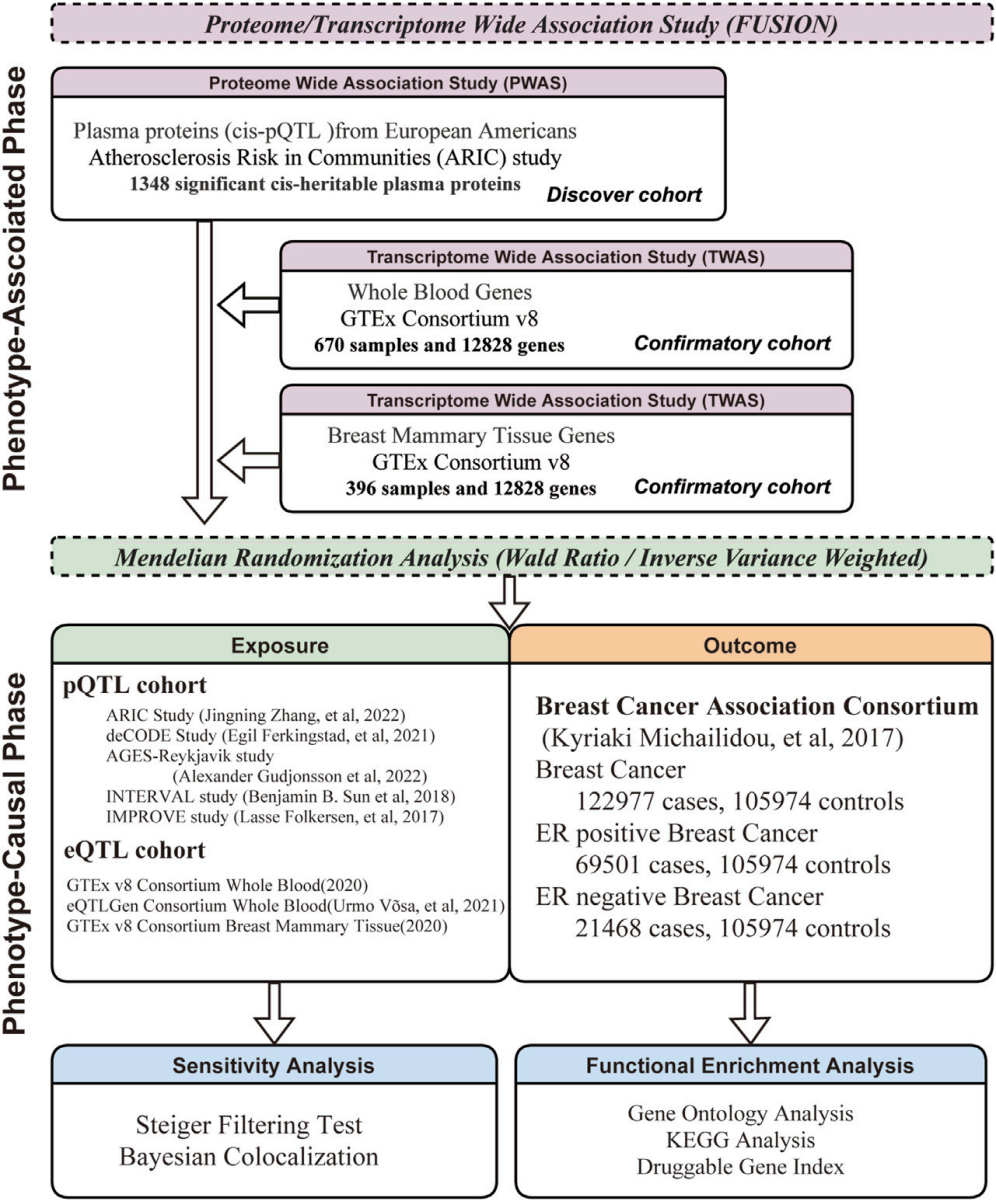
Framework of Comprehensive Research Methodology. This research methodology is divided into two phases: phenotype-association and phenotype-causation. Each phase follows a discovery-confirmatory approach.

The analysis flowchart for the study is presented in Figure 1. A two-phase analytical approach was employed in this study, merging P/TWAS for association and MR for causation. Additionally, to guarantee the validity and reliability of the findings, a discovery-confirmatory framework was implemented in both phases.

### 2.2 Breast cancer GWAS summary data source

The GWAS summary data from the Breast Cancer Association Consortium (BCAC), which specifically focused on individuals of European descent (https://bcac.ccge.medschl.cam.ac.uk/), was utilized in our study. This dataset was comprised of 122,977 breast cancer cases and 105,974 controls. The same analytical approach was also applied to ER positive and negative breast cancer. The ER-positive subtype was found to consist of 69,501 cases and 105,974 controls, while the ER-negative subtype included 21,468 cases and 105,974 controls.

### 2.3 Quantity trait loci (QTL) dataset sources

Cis-pQTL data for European Americans’ (EA) plasma proteins were obtained from the ARIC cohort (nilanjanchatterjeelab.org/pwas/), generated using PLINK2 software (Purcell et al., 2007). The SeqID file names correspond to the SOMAmers (Slow Off-rate Modified Aptamers), which are utilized for measuring protein levels in biological samples by leveraging their enhanced affinity and specificity for target proteins (Rohloff et al., 2014). For external validation, cis-pQTL data from 4 extensive plasma protein cohorts of European descent were used. Additionally, our study also explored expression quantitative trait loci (eQTLs), which influence gene expression at the transcriptome level (Zhu et al., 2016). We extracted eQTL data using the SMR toolkit (Wu et al., 2021), a tool specifically designed for genetic epidemiological research, from two major sources: the Genotype-Tissue Expression Project (GTEx Consortium, 2020) and the eQTLGen consortium (Võsa et al., 2021). Detailed descriptions of each dataset are provided in Table 1.

**TABLE 1:** Detailed information about each GWAS summary data.

GWAS	Cohort	Paper title	Year	Author	PMID	Sample size	Protein/Gene measured
pQTL							
Discovery	ARIC study	Plasma proteome analyses in individuals of European and African ancestry identify cis-pQTLs and models for proteome-wide association studies	2022	Jingning Zhang, et al	35,501,419	7,213	4435 (1,318 in PWAS)
Confirmatory	Icelandic Cancer Project (52% of participants) and deCODE genetics (48% of participants)	Large-scale integration of the plasma proteome with genetics and disease	2021	Egil Ferkingstad, et al	34,857,953	35,559	4719
Confirmatory	AGES-Reykjavik study	A genome-wide association study of serum proteins reveals shared loci with common diseases	2022	Alexander Gudjonsson, et al	35,078,996	5,368	2091
Confirmatory	INTERVAL study	Genomic atlas of the human plasma proteome	2018	Benjamin B. Sun, et al	29,875,488	3,301	2,994
Confirmatory	IMPROVE study	Mapping of 79 loci for 83 plasma protein biomarkers in cardiovascular disease	2017	Lasse Folkersen, et al	28,369,058	3,394	83
eQTL							
Confirmatory	GTEx v8 Consortium Whole Blood	The GTEx Consortium atlas of genetic regulatory effects across human tissues	2020	GTEx Consortium	32,913,098	670	12,828
Confirmatory	eQTLGen Consortium Whole Blood	Large-scale cis- and trans-eQTL analyses identify thousands of genetic loci and polygenic scores that regulate blood gene expression	2021	Urmo Võsa, et al	34,475,573	31,684	16,987
Confirmatory	GTEx v8 Consortium Breast Mammary Tissue	The GTEx Consortium atlas of genetic regulatory effects across human tissues	2020	GTEx Consortium	32,913,098	396	12,828
Breast Cancer							
Overall Breast Cancer	Association analysis identifies 65 new breast cancer risk loci		2017	Kyriaki Michailidou, et al	29,059,683	228,951	
ER positive	Association analysis identifies 65 new breast cancer risk loci		2017	Kyriaki Michailidou, et al	29,059,683	175,475	
ER negative	Association analysis identifies 65 new breast cancer risk loci		2017	Kyriaki Michailidou, et al	29,059,683	127,442	

### 2.4 Proteome/transcriptome-wide association studies with fusion

FUSION (Boston, MA, United States) (Gusev et al., 2016), which is a software to establish associations between functional phenotype and GWAS phenotype, was used to conduct P/TWAS analysis. In our study, FUSION was implemented to identify associations between protein/gene expression levels and Breast Cancer susceptibility. Methodologically, FUSION takes two inputs: 1) Precomputed functional weights, and 2) GWAS summary statistics unified to a reference SNP panel. In PWAS, precomputed functional weights of plasma proteins were obtained from the ARIC study (Zhang et al., 2022b), and the reference SNP panel was derived from the European descent of the 1000G project (http://www.internationalgenome.org/faq/how-do-i-cite-1000-genomes-project). The primary outputs of FUSION are the Z-score and *p*-value, wherein Z-score quantifies the strength and direction of the associations between plasma proteins and breast cancer, while the *p*-value elucidates the statistical significance of this association. To enhance our findings at transcriptomic level, we incorporated TWAS for both whole blood and breast mammary tissues. The precomputed functional weights for TWAS, provided by Junghyun Jung from the Mancuso lab (http://gusevlab.org/projects/fusion/). A false discovery rate (FDR, Benjamini–Hochberg) threshold of 0.05 was applied to determine the statistical significance of the results.

### 2.5 Bayesian colocalization analysis and protein association classification

Bayesian colocalization analysis (Giambartolomei et al., 2014) was utilized to evaluate the probability that the same genetic variant affects both plasma protein and breast cancer. The default parameters set by the analysis were followed, including *p*1 = 10e−4 (the probability of a variant being a significant pQTL), *p*2 = 10e−4 (the probability of a variant associated with breast cancer), and *p*12 = 10e−5 (the probability of a variant being significant in both protein/gene and GWAS). This analysis involved five predefined hypotheses: H0, indicating no association with either trait; H1, signifying association with trait1 only; H2, implying association with trait2 only; H3, representing associations with both traits due to different SNPs; and H4, indicating association with both traits due to a common SNP. A posterior probability of H4 (PPH4) exceeding 0.8, or in some cases 0.7, is generally interpreted as strong evidence of the same genetic variant being implicated in both traits (Giambartolomei et al., 2014).

Recent studies have investigated the causal associations between plasma proteins and diseases like colorectal cancer (Sun et al., 2023) and inflammatory bowel disease (Chen et al., 2023), utilizing a scoring system that integrates *p*-value and PPH4. Building on this approach, our research employs P/TWAS and Bayesian Colocalization analysis to systematically categorize the degrees of association between proteins. The scoring system was as follows: a significant adjusted *p*-value was awarded 1 point, and a PPH4 > 0.75 also earned 1 point. Based on the cumulative scores, associations were categorized as follows: a score between 1 and 2 indicated a “Weak” association, 3 to 4 suggested a “Moderate” association, and 5 to 6 signified a “Strong” association.

### 2.6 Mendelian Randomization and sensitivity analysis

In the causal analysis, we primarily conducted further analysis on proteins with strong and moderate associations. MR analysis were based on 3 essential assumptions for genetic instrumental variables: relevance, independence, and exclusion-restriction (Davies et al., 2018). We implemented a stringent selection process for SNPs to be used as instrumental variables, requiring a *p* < 5e-8, or *p* < 5e-6 in cases when SNP was absent. Clump was applied in accordance with the default parameters. The Wald Ratio (WR) method was employed when a single SNP was used as the instrumental variable, whereas the inverse-variance weighted (IVW) method was predominant when the instrumental variables involved multiple SNPs (Burgess et al., 2019). To reinforce the robustness of our findings, we conducted several sensitivity analyses. The Steiger filtering test (Deng et al., 2022) was utilized to eliminate the possibility of reverse causal associations. Additionally, heterogeneity and pleiotropy sensitivity analyses were conducted for proteins that met the criteria (Bowden et al., 2015; Greco et al., 2015). Furthermore, to improve the reliability and applicability of our results, external validation was carried out on pQTL data derived from 4 extensive plasma protein cohorts in European populations.

### 2.7 Enrichment analysis and potential druggable targets

To delve deeper into the intricate relationships and biological functions of significant proteins identified in our PWAS, gene ontology (GO) enrichment and Kyoto Encyclopedia of Genes and Genomes (KEGG) pathway analyses was performed. Given the emerging role of plasma proteins as potential therapeutic targets (Sun et al., 2018), we matched P/TWAS-MR significant proteins with the druggable genome database (Finan et al., 2017), which categorizes 4,479 genes into three druggability tiers: Tier 1 includes approved drugs and candidates in clinical trials, Tier 2 encompasses targets of biologically active molecules and those similar to approved drug targets, and Tier 3 comprises genes for secreted or extracellular proteins and other key druggable gene family members. Additionally, the significant proteins were annotated using the Therapeutic Target Database (http://db.idrblab.net/ttd/) (Zhou et al., 2022).

### 2.8 Statistical methods

In this study, data analysis was executed using R software (version 4.3.1). The P/TWAS analysis followed the analytical process previously described. The Benjamini–Hochberg method was employed for multiple testing correction, with adjusted *p*-values <0.05 considered statistically significant. Causations were investigated using the “TwoSampleMR” package, while Bayesian colocalization analysis was carried out using the “COLOC” package. The “ClusterProfiler” package (Wu et al., 2021) was utilized for functional enrichment analysis. Data visualization was achieved through the “Forestploter” and “ggplot2” packages, and data cleaning was performed using the “tidyverse” package.

## 3 Results

### 3.1 Identification of associations at the proteomic level

**FIGURE 2:**
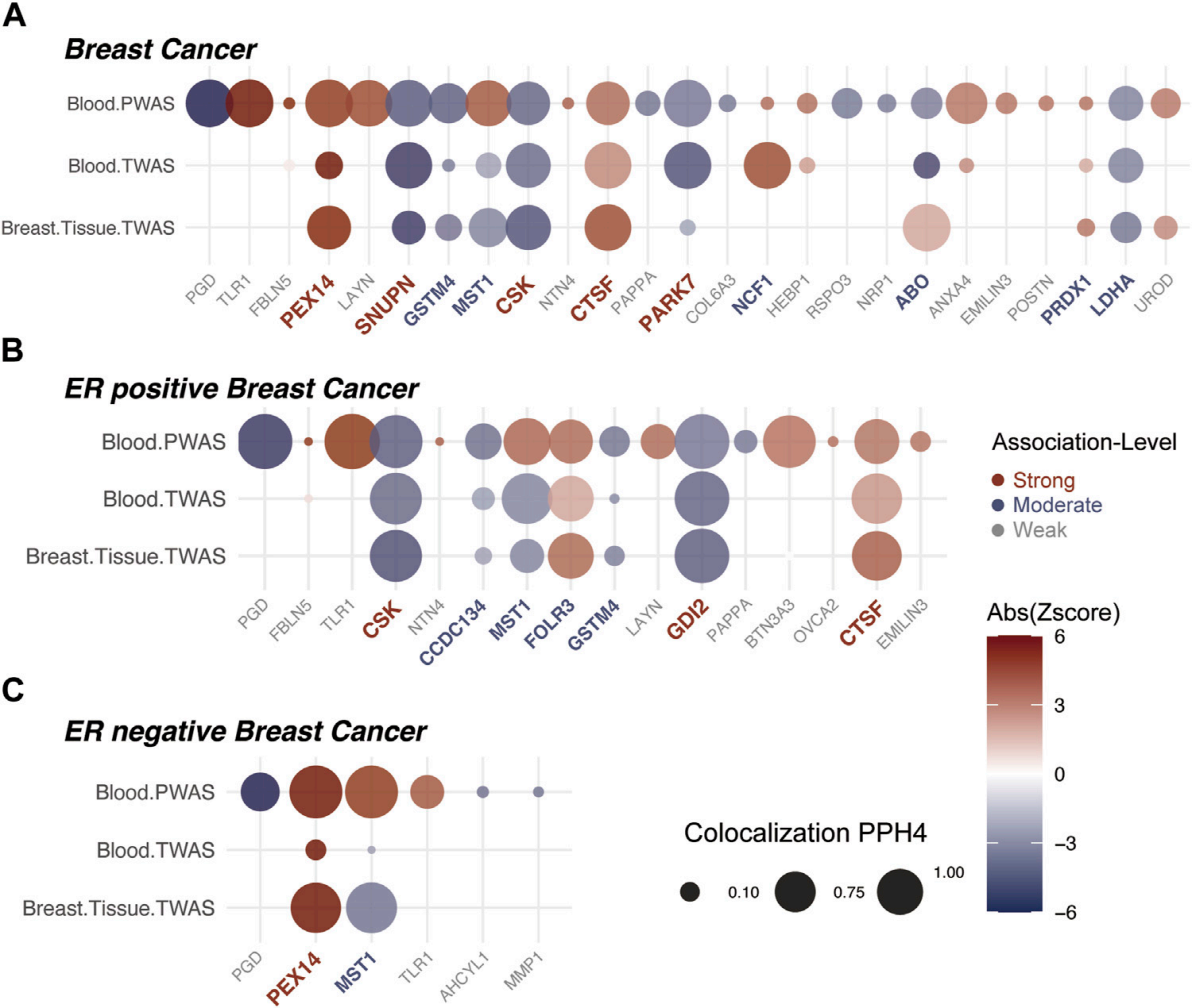
Insights from Proteome/Transcriptome-Wide Association Analyses and Bayesian Colocalization. The association strength of proteins with breast cancer is denoted by colors: red for strong, blue for moderate, and grey for weak associations. **(A)** Comprehensive P/TWAS for plasma proteins in breast cancer susceptibility. Dot size signifies results from Bayesian Colocalization analysis, with color gradient reflecting the Z-value. Proteins are sequentially arranged based on ascending *p*-value significance from left to right. **(B)** Comprehensive P/TWAS for plasma proteins in ER positive breast cancer susceptibility. **(C)** Comprehensive P/TWAS for plasma proteins in ER negative breast cancer susceptibility.

**FIGURE 3:**
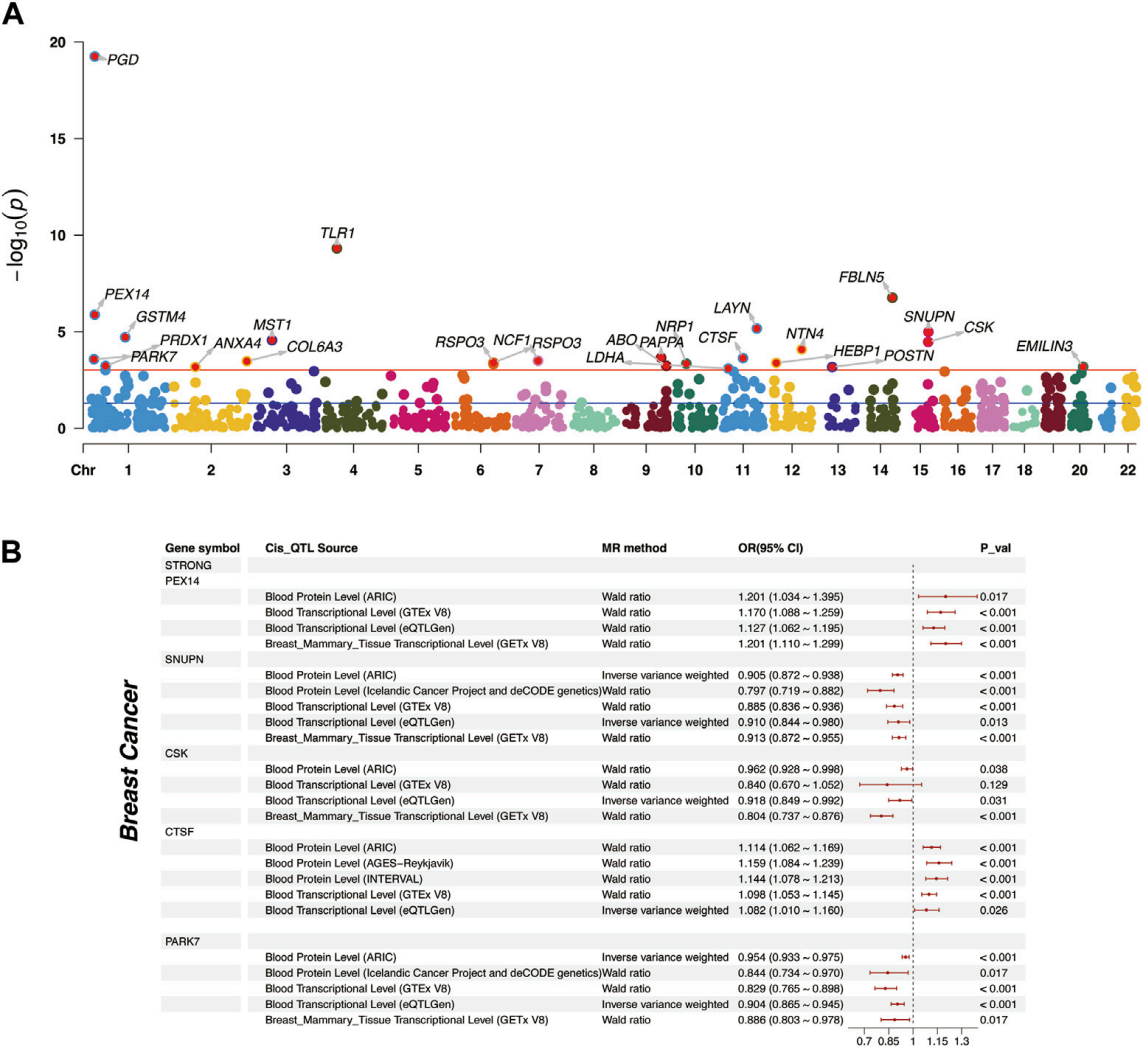
Distribution of plasma proteins and Mendelian Randomization analysis of “Strong” plasma proteins. **(A)** The Manhattan plot represented plasma proteins with significant affiliations to breast cancer. The red horizontal line indicates the FDR corrected *p*-value threshold for significance. Chromosomal designations populate the horizontal axis, contrasted with respective -log10 *p*-values on the vertical spectrum. **(B)** Two-sample Mendelian Randomization analysis for “Strong” plasma proteins to breast cancer, including external validation at proteomic and transcriptomic levels.

In our study, a total of 25 plasma proteins were significantly associated with breast cancer (Table 2; Figure 2A, and Supplementary Table S1). Of these proteins, 14 showed a Z-score greater than 0, denoting a positive association with breast cancer. Conversely, the remaining 11 proteins suggested an inverse association with the disease. When duplicate SOMAmers are present, we select the protein corresponding to the smallest *p*-value for subsequent analysis, such as RSPO3 (Supplementary Table S1). In ER subtypes analysis, 16 proteins were found to be significantly associated with ER-positive breast cancer and 6 with ER-negative breast cancer (Supplementary Table S2, S3). The PWAS Manhattan plot illustrates the distribution of significant genes across different chromosomes and their respective *p*-value (Figure 3A, Supplementary Figure S2A, B).

**TABLE 2:** Integrative analysis and stratification of proteome and transcriptome associations in breast cancer.

Gene	CHR	Plasma protein PWAS (discovery cohort)			Whole blood TWAS (confirmatory cohort)			Breast tissue TWAS (confirmatory cohort)			Score	Association power
		Zscore	*P*_FDR	PPH4	Zscore	*P*_FDR	PPH4	Zscore	*P*_FDR	PPH4		
PGD	1	−9.152	7.39E-17	0.994	-	-	-	-	-	-	2	Weak
TLR1	4	6.225	3.18E-07	1	-	-	-	-	-	-	2	Weak
FBLN5	14	5.226	7.60E-05	0	0.460	0.6460	0	-	-	-	1	Weak
PEX14	1	4.839	0.0004	0.989	6.843	1.09E-10	0.195	5.341	8.14E-07	0.79	5	Strong
LAYN	11	4.499	0.0018	0.936	-	-	-	-	-	-	2	Weak
SNUPN	15	−4.413	0.0022	0.952	−5.285	8.82E-07	0.939	−5.256	8.14E-07	0.372	5	Strong
GSTM4	1	−4.273	0.0036	0.618	−3.451	0.0011	0.001	−3.736	0.0004	0.175	3	Moderate
MST1	3	4.194	0.0045	0.904	−2.547	0.0139	0.148	−3.266	0.0015	0.584	4	Moderate (inconsistent)
CSK	15	−4.147	0.0049	0.779	−3.979	0.0002	0.843	−4.613	1.46E-05	0.863	6	Strong
NTN4	12	3.938	0.0108	0	-	-	-	-	-	-	1	Weak
PAPPA	9	−3.692	0.0255	0.137	-	-	-	-	-	-	1	Weak
CTSF	11	3.681	0.0255	0.777	2.895	0.0059	0.944	4.394	0.0000	0.939	6	Strong
PARK7	1	−3.648	0.0269	0.978	−4.644	1.20E-05	0.965	−2.391	0.0185	0.014	5	Strong
NCF1	7	3.601	0.0296	0.002	4.419	2.78E-05	0.958	-	-	-	3	Moderate
COL6A3	2	−3.585	0.0296	0.026	-	-	-	-	-	-	1	Weak
RSPO3	6	−3.541	0.0319	0.272	-	-	-	-	-	-	1	Weak
HEBP1	12	3.533	0.0319	0.058	2.358	0.0215	0.015	-	-	-	2	Weak
NRP1	10	−3.508	0.0328	0.038	-	-	-	-	-	-	1	Weak
ABO	9	−3.442	0.0369	0.298	−4.953	3.41E-06	0.169	2.127	0.0334	0.994	4	Moderate
PRDX1	1	3.436	0.0369	0.004	1.993	0.0498	0.005	3.392	0.0011	0.03	3	Moderate
EMILIN3	20	3.410	0.0369	0.072	-	-	-	-	-	-	1	Weak
ANXA4	2	3.404	0.0369	0.681	2.820	0.0067	0.008	-	-	-	2	Weak
POSTN	13	3.401	0.0369	0.01	-	-	-	-	-	-	1	Weak
LDHA	11	−3.352	0.0424	0.404	−3.307	0.0016	0.416	−3.625	0.0005	0.291	3	Moderate
UROD	1	3.302	0.0487	0.254	-	-	-	2.834	0.0056	0.113	2	Weak

### 3.2 Identification of associations at the transcriptomic level

For the 25 proteins identified by PWAS, 12 showed significant associations in the whole blood TWAS analysis (*P.adj* < 0.05) (Supplementary Table S4). While, in the breast mammary tissue TWAS, 10 of these proteins were further validated (*P.adj* < 0.05) (Table 2, Supplementary Table S5). Among the 16 significant proteins in ER-positive breast cancer, with 7 were confirmed in both whole blood and breast mammary tissue analyses. Meanwhile, in ER-negative breast cancer, 2 out of the 6 significant proteins were validated (Figures 2B,C, Supplementary Table S6). It should be noted that MST1 exhibited contradictory associations in PWAS (Z = 4.194, *P.adj* = 0.004) and TWAS (Z = −2.547, *P.adj* = 0.014). This pattern was also observed in ER-positive and ER-negative subtypes. Due to the complex nature and potential biological implications of MST1’s contrasting results, we did not conduct further analysis on this protein.

### 3.3 Bayesian Colocalization analysis

Among 25 significant proteins, 9 exhibited strong genetic colocalization evidence. Additionally, 4 proteins–SNUPN (PPH4 = 93.9%), CSK (PPH4 = 84.3%), CTSF (PPH4 = 94.4%), and PARK7 (PPH4 = 96.5%)–also demonstrated the same strong genetic evidence at the whole blood transcriptomic level. Remarkably, CSK (PPH4 = 86.3%) and CTSF (PPH4 = 93.9%) were further validated in the breast mammary tissue transcriptomic level (Table 2). In ER-positive breast cancer, 5 proteins showed strong evidence of genetic colocalization. Notably, 2 of these proteins, CSK (PPH4 = 85.8%, 86.5%) and GDI2 (PPH4 = 97%, 97.2%), demonstrated the same strong genetic colocalization evidence in both whole blood and breast mammary tissues. In the ER-negative breast cancer, PEX14 showed strong genetic colocalization evidence in protein (PPH4 = 99.9%) and breast mammary tissue (PPH4 = 88.8%), but this pattern was not replicated at the whole blood transcriptomic level (PP4 = 8%, Supplementary Table S6).

### 3.4 Stratification of plasma protein association strengths

In breast cancer, 25 proteins were classified: 5 as “Strong” association (red), 6 as “Moderate” association (blue), and the remaining as “Weak” association (grey) (Figure 2A). Among the “Strong” Tiers, PEX14 (Z = 4.839) and CTSF (Z = 3.681) had a positive association with breast cancer. Whereas, SNUPN (Z = −4.413), CSK (Z=−4.417), and PARK7 (Z = −3.648) showed negative associations (Table 2).

In ER-positive breast cancer, 16 proteins were classified: 3 proteins showed “Strong” association (red), with GDI2 (Z = −3.652) newly identified and negatively associated with ER-positive breast cancer. Additionally, CSK and CTSF followed the same trends with the findings from breast cancer. Besides, 4 proteins were “Moderate” (blue), and 9 proteins were “Weak” associations (grey) (Figure 2B). In ER-negative breast cancer, 6 proteins were classified: PEX14 and MST1 showed “Strong” associations. Notably, PEX14 not only showed the same trend as observed in breast cancer (Z = 4.839, *p =* 0.0004) but also exhibited a notably stronger effect (Z = 5.929, *p =* 2.02E-6). MST1 was not further analyzed due to inconsistent trends in P/TWAS. The other 4 proteins were categorized as “Weak” association (grey) (Figure 2C, Supplementary Table S6).

It is crucial to highlight that, although PGD and TLR1 were significant across all three outcomes in PWAS analyses (Figure 3A and Supplementary Figure S2A, B), their absence from the corresponding TWAS analysis relegated them to the “Weak” association. Moreover, the results of these two proteins were not sufficiently reliable in MR Analysis (Supplementary Figure S2C, Supplementary Table S7).

### 3.5 Mendelian Randomization analyses

Upon determining the strength of associations, we supplemented the causations with MR analysis (Supplementary Table S8). We primarily focused on the causal effects of “Strong” associated proteins. Among the 5 “Strong” associated proteins, PEX14 was found to have a positive causation at the proteomic (OR = 1.201, *p* = 0.017) and transcriptomic level (OR = 1.17, *p* < 0.001). CTSF demonstrated a positive causation in the ARIC cohort (OR = 1.114, *p* < 0.001), and the consistent trends were also external validated in INTERVAL cohort (OR = 1.144, *p* < 0.001) (Sun et al., 2018) and AGES-Reykjavik cohort (OR = 1.159, *p* < 0.001) (Gudjonsson et al., 2022) (Figure 3B). The remaining 3 proteins, SNUPN (OR = 0.905, *p* < 0.001), CSK (OR = 0.962, *p* = 0.038), and PARK7 (OR = 0.954, *p* < 0.001), all exhibited negative causations with breast cancer. External validations from the deCODE cohort further confirmed the causations for SNUPN (OR = 0.797, *p* < 0.001) and PARK7 (OR = 0.844, *p* = 0.017). However, CSK’s causation at the whole blood transcriptomic level was somewhat unsignificant (OR = 0.84, *p* = 0.129) (Figure 3B, Supplementary Table S9).

**FIGURE 4:**
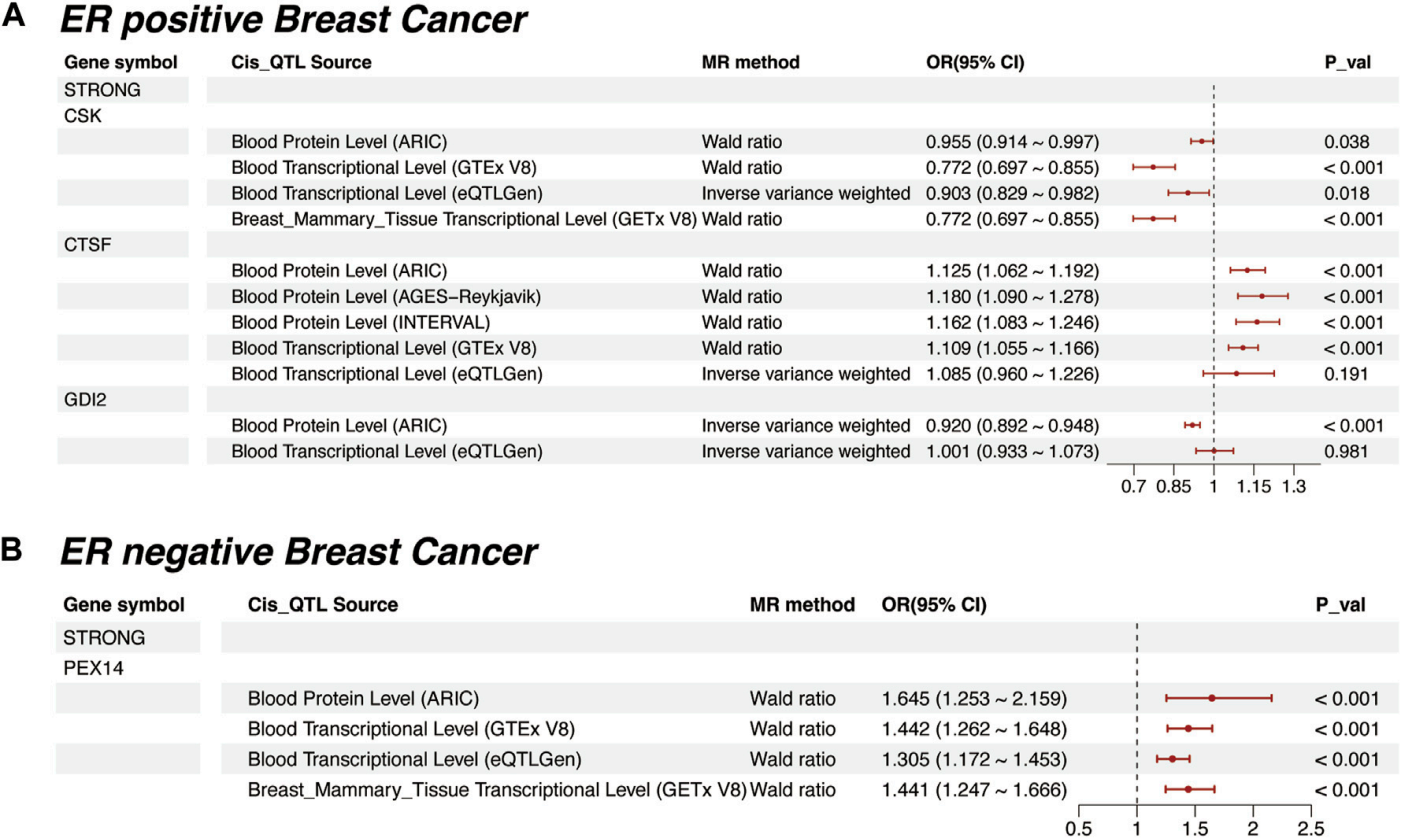
Mendelian Randomization for “Strong” plasma proteins in different ER Breast Cancer Subtypes. **(A)** Mendelian randomization results for ER-positive breast cancer, including external validation at proteomic and transcriptomic levels. **(B)** Mendelian randomization results for ER-negative breast cancer, including external validation at proteomic and transcriptomic levels.

In ER-positive breast cancer, CSK (OR = 0.955, *p* = 0.038) and CTSF (OR = 1.125, *p* < 0.001) maintained the same causal trends as observed in breast cancer (Supplementary Table S10). Additionally, GDI2 was identified as a newly negatively significant protein (OR = 0.92, *p* < 0.001). However, its causal effect was not significant at the transcriptomic level (OR = 1.001, *p* < 0.981, Figure 4A). In ER-negative breast cancer, PEX14 stood out as the sole “Strong” protein. Notably, its causal effect in this subtype (OR = 1.645, *p* < 0.001, Figure 4B) was further pronounced compared to breast cancer (OR = 1.201, *p* = 0.017). Meanwhile, we expanded our MR analyses to include “Moderate” proteins. The results revealed that their causal effects were generally less consistent and of reduced significance compared to those of the “Strong” proteins (Supplementary Figure S1, Supplementary Table S11).

### 3.6 Sensitivity analysis and functional enrichment analysis

Considering that the pQTLs of most plasma proteins was a single SNP, conducting sensitivity analyses for heterogeneity and pleiotropy is typically not required. As result, in ER-positive breast cancer, BTN3A3, EMILIN3, FOLR3, and NTN4 showed heterogeneity, while in ER-negative cases, this was not observed (Supplementary Table S10). BTN3A3 in ER-positive breast cancer also displayed pleiotropy. The Steiger filtering test confirmed that MR effects were due to plasma proteins affecting breast cancer outcomes (Supplementary Table S8, S10). Importantly, our “Strong” proteins exhibited neither heterogeneity nor pleiotropy.

Furthermore, the plasma proteins identified by PWAS were subjected to Gene Ontology (GO) cluster analysis. This analysis revealed a predominant association with biological processes related to oxidative stress, such as “reactive oxygen species metabolic” and “response to reactive oxygen species” terms. Additionally, for cellular components, we observed a significant enrichment in the “collagen-containing extracellular matrix” term (Supplementary Figure S3). Besides, KEGG pathway enrichment did not reveal any significantly enriched pathways (Supplementary Table S12).

### 3.7 Druggable target propensity for significant proteins

Plasma proteins are not only crucial as diagnostic biomarkers but also serve as potential drug targets. In our study, we evaluated the significant proteins for their potential as drug targets. By aligning our findings with the druggable genome database (Finan et al., 2017), we determined that 16 of the 25 proteins have druggable targets. These include 3 proteins in Tier 1; 3 in Tier 2, and 10 in Tier 3 (Supplementary Table S13, Left column). Furthermore, we compared our results with the Therapeutic Target Database (Zhou et al., 2022), 11 of these 16 proteins were identified as targets of existing or potential drugs. This group comprised 3 Successful targets, 3 Patented-recorded Targets, 1 in clinical trials, and 4 documented in literature (Supplementary Table S13 Right column). Among the “Strong” proteins, CSK and CTSF were found to be drug targets with patent records, categorized under Tiers 1 and 2 respectively. CTSF has been documented to be used in the treatment of bone cancer and chronic obstructive pulmonary disease (Li et al., 2017) (Table 3). However, the remaining “Strong” proteins have not yet been reported.

**TABLE 3:** Comprehensive evaluation of strong associated proteins as potential druggable targets or existing therapeutics.

Gene	UniProt	Description	Finan et al	Therapeutic target database		
			Tier	Target type	Drug name	Disease
Breast Cancer						
PEX14	O75381	Peroxisomal Biogenesis Factor 14	-	-	-	-
SNUPN	O95149	Snurportin 1	-	-	-	-
CSK	P41240	Tyrosine-protein kinase CSK	Tier 1	Patented-recorded Target	936,563-93-8	Not Available
CTSF	Q9UBX1	cathepsin F	Tier 2	Patented-recorded Target	PMID27998201-Compound-5	Bone cancer; Chronic obstructive pulmonary disease
PARK7	Q99497	Parkinsonism Associated Deglycase	-	-	-	-
ER pos Breast Cancer						
CSK	P41240	Tyrosine-protein kinase CSK	Tier 1	Patented-recorded Target	936,563-93-8	Not Available
CTSF	Q9UBX1	cathepsin F	Tier 2	Patented-recorded Target	PMID27998201-Compound-5	Bone cancer; Chronic obstructive pulmonary disease
GDI2	P50395	Rab GDP dissociation inhibitor beta	-	-	-	-
ER neg Breast Cancer						
PEX14	O75381	Peroxisomal Biogenesis Factor 14	-	-	-	-

## 4 Discussion

Plasma proteins, due to their ease of detection and reproducibility, are increasingly utilized to distinguish between cancer patients and healthy individuals, enhancing the effectiveness of screening programs (Huijbers et al., 2010). Recent advancements in molecular technologies and techniques have shown significant potential in utilizing plasma protein biomarkers such as Adipsin and CA15-3 for early detection and quantification for diagnostic and therapeutic applications in breast cancer (Afzal et al., 2022; Rajkumar et al., 2022; Veyssière et al., 2022). A recent high-throughput study identified 61 proteins associated with various cancers (Gregga et al., 2023). While this study provided valuable insights into pan-cancer associations, it did not explore causation. Furthermore, research specifically targeting plasma protein biomarkers for breast cancer is still limited. Currently, Mendelian Randomization has emerged as an effective method to establish causation in various diseases (Emdin et al., 2017), including cholesterol-related cardiovascular disease (Kathiresan et al., 2008), inflammatory diseases (Swerdlow et al., 2012), metabolic disorders (Fall et al., 2015), and specific cancers such as small cell lung cancer and colorectal cancer (Sun et al., 2023; Wu et al., 2023).

Despite, the application of MR in identifying plasma proteins as drug targets in breast cancer is still sporadic. For instance, one study performed MR analysis on a single cohort of 732 plasma proteins, where GDI2 and CTSF were identified as potential targets for breast cancer (Ren et al., 2023), aligning with our research. However, it is important to note that this study also focused on pan-cancer research and lacked association analysis. Additionally, another study focused on the causation found a causal link between TLR1 and breast cancer (Mälarstig et al., 2023). This protein was ranked significantly in our analysis, but it is noteworthy that TLR1 lacks external cohort validation, and the study also did not perform association analyses. Therefore, current research on plasma proteins typically focuses on either association or causation, rarely addressing both. Our study bridges this gap by integrating these two approaches. We employed P/TWAS to identify associations and used MR to establish causation. This approach successfully pinpointed significant proteins related to breast cancer risk from thousands of candidates in 5 large proteomics cohorts. To ensure the robustness and generalizability of our findings, we adopted a “discovery-confirmatory” analytical framework at both the association and causation phases. Overall, we found 5 proteins (PEX14, CTSF, SNUPN, CSK, PARK7) with strong causal links to breast cancer. While, in ER-positive breast cancer, 3 proteins (CSK, CTSF, GDI2) were identified. In contrast, only PEX14 was linked to ER-negative breast cancer.

Among the 5 plasma proteins, SNUPN, CSK, and PARK7 emerged as “Strong” negatively causative associated proteins, indicating a protective effect against breast cancer development. A study has highlighted the potential clinical applications of SNUPN in acute lymphoblastic leukemia (Mata-Rocha et al., 2019); however, research exploring its role in solid tumors, including breast cancer, is currently limited. Despite the current research limitations, SNUPN’s potential as a biomarker or tumor suppressor is promising and warrants further exploration. PARK7 is recognized for its neuroprotective role in Parkinson’s disease (Kochmanski et al., 2022) and has been reported to significantly regulate cell survival and cancer progression in various cancers (Jin, 2020). It negatively regulates PTEN and PKB/Akt phosphorylation, thus influencing cell survival and death (Kim et al., 2005). In breast cancer, low PARK7 expression was correlated with pathological complete response in 79.6% of cases following neoadjuvant therapy (Kawate et al., 2013), and loss of PARK7 function is associated with increased sensitivity to doxorubicin in breast cancer cells (Zhang et al., 2015). The effect of PARK7 in balancing tumor cell survival and normal cell physiology merits further research. Lastly, as a key member of the Src family kinases (SFKs), CSK plays a vital role in combating cancer progression in various cancers (Sabe et al., 1994). Recent study indicates that CSK maintains negative regulation of Src through Tyr527 phosphorylation, inhibiting breast cancer cells growth and spread (Dias et al., 2022). Additionally, another study on ER-positive breast cancer found that in cases of endocrine therapy resistance, reduced CSK leads to enhanced PAK2 activity and subsequent non-estrogen-dependent cancer growth (Xiao et al., 2018). The dual effect of CSK in both tumor suppression and inducing endocrine treatment resistance positions it as a notable target for research.

The other two “Strong” proteins are positively associated and represent a risk factor in breast cancer onset. CTSF (cathepsin F) plays a key role in the lysosomal protein degradation pathway (Wex et al., 1999). Currently, it is reported as an effective diagnostic biomarker in cervical cancer (Vazquez-Ortiz et al., 2005), gastric cancer (Ji et al., 2018), and non-small cell lung cancer (Wei et al., 2022). A recent study reported that CTSF may act as an independent poor prognostic factor for basal-like breast cancer (Huang et al., 2021). PEX14 (Peroxisomal Biogenesis Factor 14) is essential for peroxisomal biogenesis (Neufeld et al., 2009). Our research reveals a significant causal risk association of PEX14 with breast cancer (OR = 1.201), particularly in ER-negative subtype (OR = 1.645). Notably, PEX14 has been identified as a key risk factor in triple-negative breast cancer (TNBC) (Purrington et al., 2014) and is one of the top five genes influencing adaptive anti-tumor immunity, as shown in a TNBC model study using a whole-genome RNAi screening platform (Shuptrine et al., 2017). These insights emphasize PEX14’s importance in TNBC immunotherapy and drug target research. Furthermore, PEX14 plays a crucial role in maintaining peroxisomal functions, and its deficiency leads to ROS accumulation, lipid peroxidation, and consequent cell death (Guo et al., 2023). Our functional enrichment analysis corroborates this, highlighting numerous pathways related to reactive oxygen species (ROS), which are instrumental in promoting cell growth, cancer progression, immune responses, and poorer survival outcomes in breast cancer (Oshi et al., 2022). Additionally, studies have shown that PEX14 knockdown increases intracellular H_2_O_2_ levels, triggering ferroptosis and cell death (Guan et al., 2022). This further underscores PEX14’s pivotal role in managing oxidative stress and cell viability, marking its significance in breast cancer research. Additionally, GDI2 was identified as a protein with a “Strong” negative causal association in the ER-positive breast cancer. A study suggested that GDI2 is associated with aggressive features and poor patient survival in hepatocellular carcinoma (Zhang et al., 2021). However, the inability to confirm its role through at additional transcriptomic levels and the absence of external validation has diminished our confidence in the significance of this protein.

Given the proven effectiveness of MR in identifying drug targets (Folkersen et al., 2020), we performed a drug-target evaluation on these plasma proteins (Supplementary Table S13). Notably, CSK and CTSF emerged as Tier1 and Tier2 proteins, respectively. CSK is crucial in regulating cellular processes such as apoptosis, survival, and proliferation. Its pivotal role in cancer cell signaling earmarks CSK as a promising target for cancer therapy (Fortner et al., 2022). Similarly, CTSF, known for its significant involvement in the progression of various cancers (Wei et al., 2022), neurodegenerative diseases (van der Zee et al., 2016), and skin aging (Takaya et al., 2023), garners attention. Research on inhibitors and modulators targeting CTSF is underway. Although other strongly associated proteins currently lack clear therapeutic applications, given their strong causal relationship with breast cancer, it is worthwhile to further explore them for drug target development.

This study is currently subject to several limitations yet. First, the study only involves individuals of European descent, which necessitates caution when applying these findings to more diverse populations. Second, the precomputed functional weights for plasma proteins are currently only available from the ARIC cohort, future datasets expansion are expected to enhance the precision and breadth of such analyses. In addition, as the current BCAC molecular subtype data lacks rsID, matching chromosomes and base pair positions results in significant information loss. However, with the continuous expansion and updating of the molecular subtype database, we anticipate a deeper understanding of this content. Lastly, our analysis is primarily data-based, hence we will design related basic scientific research in the future to further investigate the etiological association between plasma proteins and breast cancer.

In summary, our study successfully identified several plasma proteins with strong association and causation to breast cancer and its distinct ER subtypes. As non-invasive and dynamic monitoring tools, plasma proteins hold significant potential as diagnostic biomarkers and therapeutic targets. They offer a comprehensive perspective on systemic health, which is crucial for early tumor detection, assessing treatment responses, and continuous disease monitoring. While these advancements are still in the early stages, they hold valuable promise for future research and practical applications in real-world scenarios.

## Data Availability

The original contributions presented in the study are included in the article/Supplementary Materials, further inquiries can be directed to the corresponding authors.
