# Supplementary material for: Elucidating the susceptibility to breast cancer: an in-depth proteomic and transcriptomic investigation into novel potential plasma protein biomarkers
Source: Front Mol Biosci. 2024 Jan 18;10:1340917. doi: 10.3389/fmolb.2023.1340917 (PMC10833003; doi:10.3389/fmolb.2023.1340917)
Supplement: Supplementary file 1 [file DataSheet1.zip › Supplementary_Figures_and_tables_Legends_Frontiers_Template_submission_revision.docx]

Supplementary Materials

# Supplementary Figures Legends

**Supplementary Figure 1** Comprehensive Mendelian Analysis of “Moderate” Plasma Proteins. (A) Two-sample Mendelian Randomization analysis for “Moderate” plasma proteins in ER positive breast cancer, including external validation at proteomic and transcriptomic levels. (B) Two-sample Mendelian Randomization analysis for “Moderate” plasma proteins in ER negative breast cancer, including external validation at proteomic and transcriptomic levels.

**Supplementary Figure 2** Distribution of plasma proteins identified by ER positive and negative breast cancer PWAS analysis. Chromosomal designations populate the horizontal axis, contrasted with respective -log10 *P*-values on the vertical spectrum. The red horizontal line indicates the FDR corrected *P*-value threshold for significance. (A) The Manhattan plot represented plasma proteins with significant affiliations to ER positive breast cancer. (B) The Manhattan plot represented plasma proteins with significant affiliations to ER negative breast cancer. (C) Two-sample Mendelian Randomization analysis for PGD and TLR1, including external validation at proteomic and transcriptomic levels.

**Supplementary Figure 3** Enrichment analysis of plasma proteins identified by PWAS. Gene Ontology (GO) enrichment articulates pathways intricately associated with cellular oxidative response.

# Supplementary Tables

**Supplementary Table 1 (Table S1)**: Comprehensive and Detailed Proteome-Wide Association Study (PWAS) Results on Plasma Proteins Associated with Breast Cancer.

**Supplementary Table 2 (Table S2)**: Comprehensive and Detailed Proteome-Wide Association Study (PWAS) Results on Plasma Proteins Associated with ER positive Breast Cancer.

**Supplementary Table 3 (Table S3)**: Comprehensive and Detailed Proteome-Wide Association Study (PWAS) Results on Plasma Proteins Associated with ER negative Breast Cancer.

**Supplementary Table 4 (Table S4)**: Comprehensive and Detailed Transcriptome-Wide Association Study (TWAS) Results on Whole Blood Associated with Breast Cancer and its ER subtypes.

**Supplementary Table 5 (Table S5)**: Comprehensive and Detailed Transcriptome-Wide Association Study (TWAS) Results on Breast Mammary Tissue Associated with Breast Cancer and its ER subtypes.

**Supplementary Table 6 (Table S6)**: Integrative Analysis and Categorization of Proteome and Transcriptome Associations in ER positive and negative Breast Cancer.

**Supplementary Table 7 (Table S7)**: Mendelian Randomization Analysis of PGD and TLR1.

**Supplementary Table 8 (Table S8)**: Mendelian Randomization Analysis of the ARIC protein cohort to Breast Cancer.

**Supplementary Table 9 (Table S9)**: External Validation and Detailed Analysis of plasma proteins and gene expression in Breast Cancer for Strong and Moderate Association Groups.

**Supplementary Table 10 (Table S10)**: Mendelian Randomization Analysis of the ARIC plasma protein cohort to ER positive/negative Breast Cancer.

**Supplementary Table 11 (Table S11)**: External Validation and Detailed Analysis of plasma proteins and gene expression in ER positive/negative Breast Cancer for Strong and Moderate Association Groups.

**Supplementary Table 12 (Table S12)**: KEGG results of PWAS significant protein in Breast Cancer.

**Supplementary Table 13 (Table S13)**: Comprehensive Evaluation of Proteins as Potential Druggable Targets or Existing Therapeutics.
