# Supplementary figures and images for: Elucidating the susceptibility to breast cancer: an in-depth proteomic and transcriptomic investigation into novel potential plasma protein biomarkers
Source: Front Mol Biosci. 2024 Jan 18;10:1340917. doi: 10.3389/fmolb.2023.1340917 (PMC10833003; doi:10.3389/fmolb.2023.1340917)

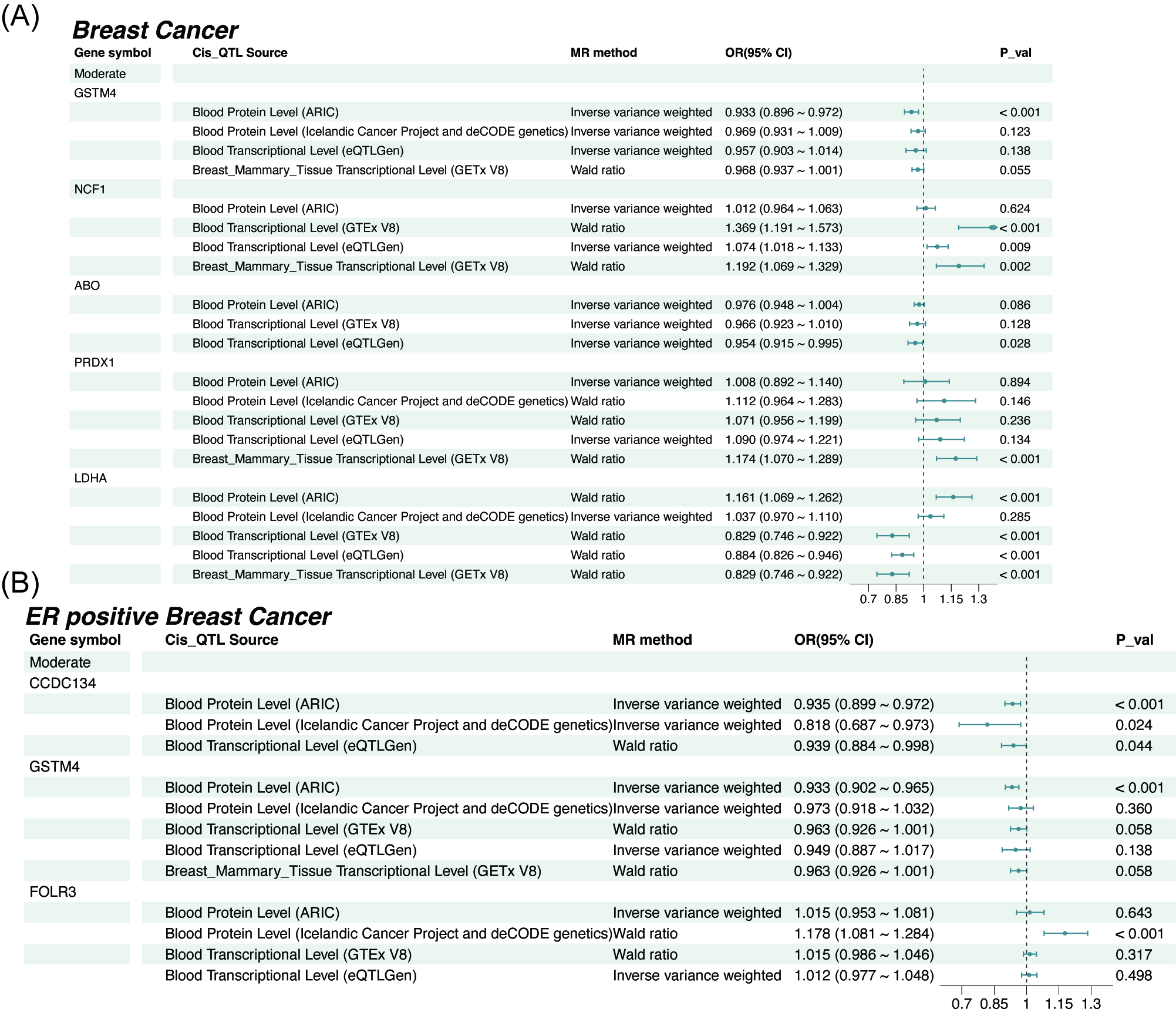

Supplement: Supplementary file 1 [file DataSheet1.zip › supplementary Figure 1.tif]

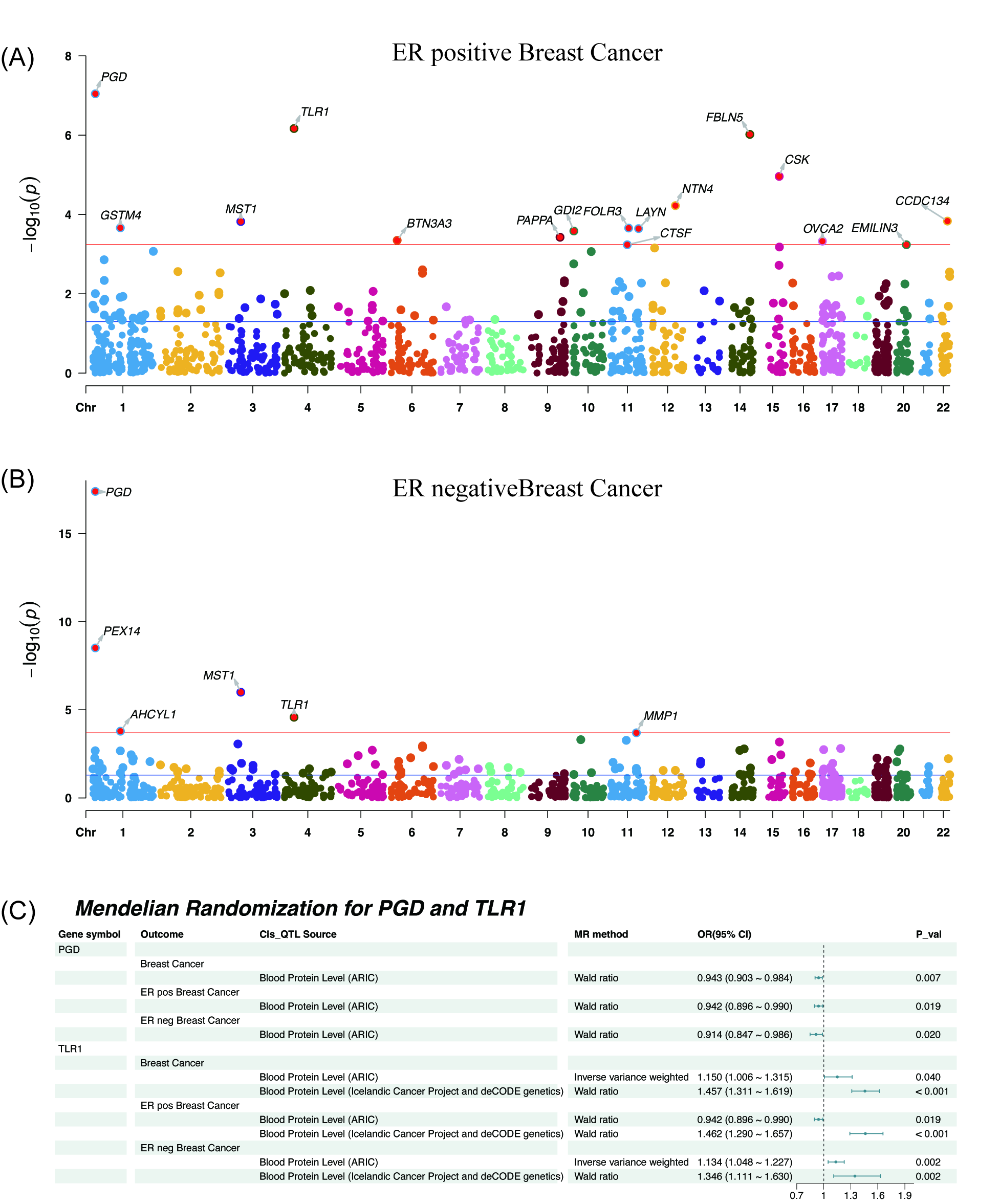

Supplement: Supplementary file 1 [file DataSheet1.zip › supplementary Figure 2.tif]

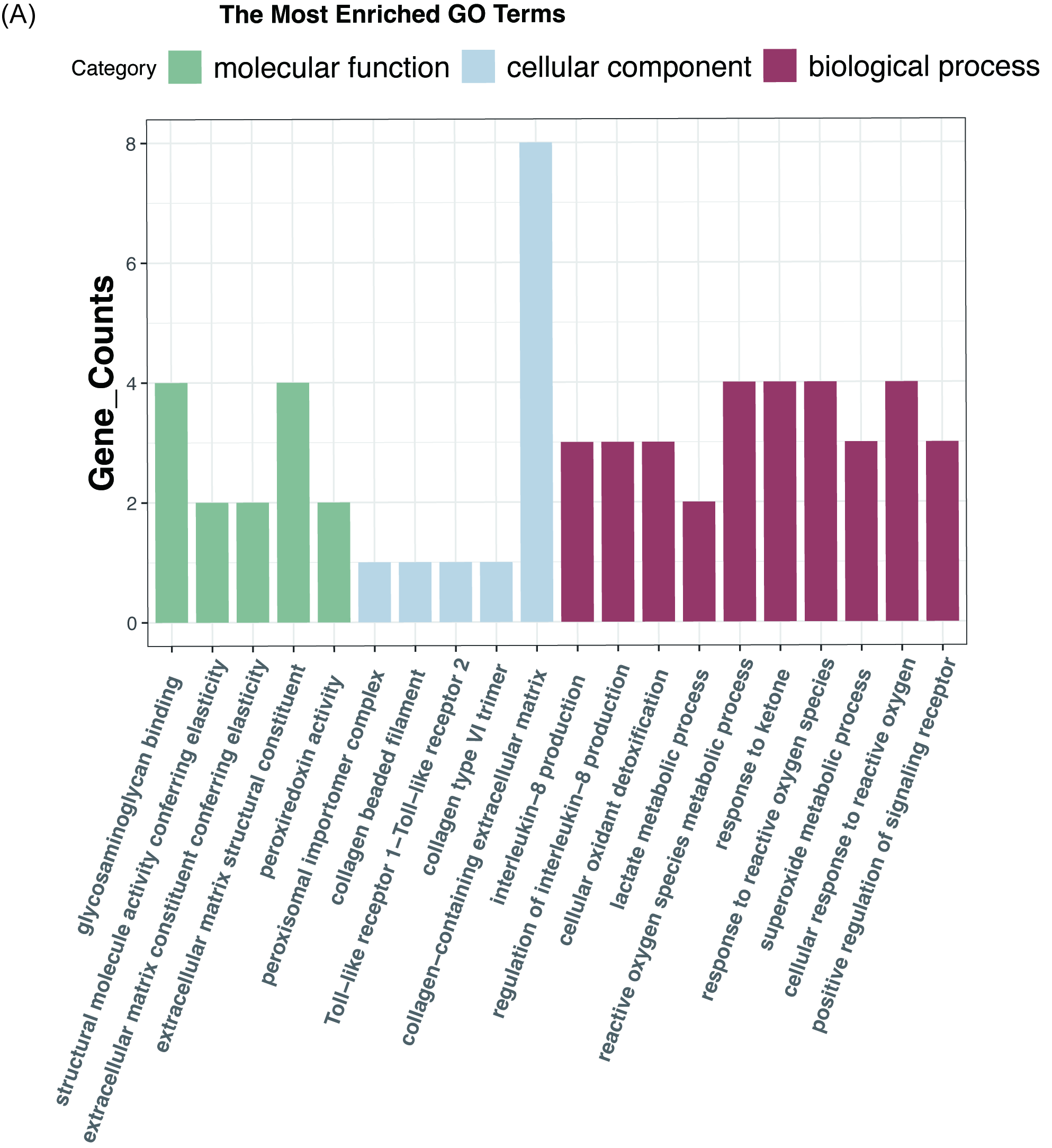

Supplement: Supplementary file 1 [file DataSheet1.zip › supplementary Figure 3.tif]
